# Nicotinic acetylcholine receptors mediate lung cancer growth

**DOI:** 10.3389/fphys.2013.00251

**Published:** 2013-09-17

**Authors:** Ma. Reina Improgo, Lindsey G. Soll, Andrew R. Tapper, Paul D. Gardner

**Affiliations:** Department of Psychiatry, Brudnick Neuropsychiatric Research Institute, University of Massachusetts Medical SchoolWorcester, MA, USA

**Keywords:** nicotinic acetylcholine receptor, ligand-gated ion channel, lung cancer, small cell lung carcinoma, CHRNA5

## Abstract

Ion channels modulate ion flux across cell membranes, activate signal transduction pathways, and influence cellular transport—vital biological functions that are inexorably linked to cellular processes that go awry during carcinogenesis. Indeed, deregulation of ion channel function has been implicated in cancer-related phenomena such as unrestrained cell proliferation and apoptotic evasion. As the prototype for ligand-gated ion channels, nicotinic acetylcholine receptors (nAChRs) have been extensively studied in the context of neuronal cells but accumulating evidence also indicate a role for nAChRs in carcinogenesis. Recently, variants in the nAChR genes *CHRNA3, CHRNA5*, and *CHRNB4* have been implicated in nicotine dependence and lung cancer susceptibility. Here, we silenced the expression of these three genes to investigate their function in lung cancer. We show that these genes are necessary for the viability of small cell lung carcinomas (SCLC), the most aggressive type of lung cancer. Furthermore, we show that nicotine promotes SCLC cell viability whereas an α3β4-selective antagonist, α-conotoxin AuIB, inhibits it. Our findings posit a mechanism whereby signaling via α3/α5/β4-containing nAChRs promotes lung carcinogenesis.

## Introduction

Lung cancer remains the leading cause of cancer-related deaths worldwide (WHO, 2011). Despite considerable research efforts to elucidate the molecular underpinnings of the disease, the 5-year survival rate for lung cancer has not changed appreciably over the past three decades and persists at a dismal 15%. The two major types of lung cancer are non-small cell lung carcinoma (NSCLC) and SCLC. The former consists of a heterogeneous group of tumors that account for 80% of lung cancer cases while the latter is less common (15–20% of cases) but is particularly aggressive (Rom et al., 2000; Sandler, 2003). SCLC is characterized by rapid growth and early dissemination resulting in an extremely poor prognosis for which no effective treatments are currently available (Rudin et al., 2008).

Cigarette smoking is the major risk factor associated with lung cancer. This is not surprising given that tobacco contains ~250 damaging chemicals and ~50 carcinogens (Hecht, 1999). In the United States alone, over 45 million adults continue to smoke while globally, 10 million cigarettes are sold every minute, making tobacco use the leading cause of preventable deaths (WHO, 2011). Prevention efforts are hampered, however, by the strong reinforcing effects of nicotine, the primary psychoactive component in tobacco.

Nicotine's effects are mediated by nAChRs that are expressed in the reward circuitry and other areas of the brain (Albuquerque et al., 2009). nAChRs are also activated by the endogenous ligand acetylcholine (ACh), hence their name. Additionally, nAChRs are activated by the nitrosamine 4-(methylnitrosamino)-1-(3-pyridyl)-1-butanone (NNK), the most potent carcinogen in tobacco (Schuller, 2007). Receptor activation allows the flow of sodium, potassium and calcium ions down their electrochemical gradients.

nAChRs are composed of transmembrane subunits that share a common evolutionary origin (Le Novere et al., 2002). In mammalian systems, these subunits are encoded by eleven genes located across different chromosomes (Table 1). The genes encoding the human α3, α5, and β4 subunits are found in a gene cluster in chromosome 15q24 and are thought to be both independently and coordinately regulated (Boulter et al., 1990; Scofield et al., 2008). The α3 subunit is usually co-expressed with the β4 subunit while α5 serves as an auxiliary subunit, whose incorporation modifies the calcium permeability of the receptor and its affinity to and desensitization by agonists (Ramirez-Latorre et al., 1996; Yu and Role, 1998). α3β4-containing nAChRs exhibit lower affinity for nicotine and are less desensitized by it, suggesting that this receptor subtype may mediate nicotine's rewarding effects after high affinity nAChR subtypes have been desensitized (Paradiso and Steinbach, 2003; Rose, 2007). Moreover, α3β4α5 nAChRs are thought to play a role in nicotine withdrawal and consistently, are highly expressed in brain regions associated with nicotine withdrawal, such as the medial habenula and the interpeduncular nucleus (Damaj et al., 2003; Salas et al., 2009).

**Table 1:** **Chromosomal locations of genes encoding nAChR subunits**.

Subunit^*^	**Gene**	Chromosome location^**^		
		**Mouse**	**Rat**	**Human**
α2	*CHRNA2*	14	15p12	8p21
α3	*CHRNA3*	9	8q24	15q24
α4	*CHRNA4*	2	3q43	20q13.2-q13.3
α5	*CHRNA5*	9	8q24	15q24
α6	*CHRNA6*	8	16q12.3	8p11.21
α7	*CHRNA7*	7	1q22	15q14
α9	*CHRNA9*	5	14p11	4p14
α10	*CHRNA10*	7	1q32	11p15.5
β2	*CHRNB2*	3	2q34	1q21.3
β3	*CHRNB3*	8	16q12.3	8p11.2
β4	*CHRNB4*	9	8q24	15q24

* α8 is expressed only in avian species.

** Summarized from the Entrez Gene Database (Maglott et al., 2011).

Multiple genome-wide association studies (GWAS) have implicated the *CHRNB4/A3/A5* locus in nicotine dependence and lung cancer (Amos et al., 2008; Hung et al., 2008; Thorgeirsson et al., 2008). A particularly interesting variant in this locus is the non-synonymous single nucleotide polymorphism (SNP) that lies in the fifth exon of *CHRNA5* (rs16969968). This variant encodes a change from an aspartic acid to an asparagine residue at amino acid position 398 (D398N). The asparagine risk allele is associated with decreased maximal response to agonists, indicating altered receptor function (Bierut et al., 2008; George et al., 2012). Additionally, the genotype in this locus appears to correlate with mRNA levels suggesting that rs16969968 may influence *CHRNA5* expression as well (Falvella et al., 2009; Wang et al., 2009). In α3β4α5 nAChRs, the 398 residue also lies close to a β4 residue that is necessary for β 4′s ability to increase nicotine-evoked currents, which subsequently leads to nicotine aversion (Frahm et al., 2011). Notably, this increase in current is maximally competed by the D398N variant, resulting in reversal of nicotine aversion. Altogether, these results support the functional relevance of the rs16969968 variant.

The association of nAChR variants with both nicotine dependence and lung cancer susceptibility prompts two hypotheses regarding the role of nAChRs in lung cancer. One hypothesis is that nicotine mainly influences nAChRs in the brain, such that increased levels of nicotine dependence consequently lead to greater exposure to tobacco carcinogens and to lung cancer development (Le Marchand et al., 2008). Consistently, α3, α5, and β4 nAChR subunits are predominantly expressed in select neural circuits that control nicotine intake in rodent nicotine dependence models (Fowler et al., 2011; Frahm et al., 2011). An alternative hypothesis is that the association between nAChR variants and lung cancer is direct, in that altered nAChR function, as encoded by risk alleles, promote carcinogenic processes in the lungs and airway tissues (Schuller, 2009). The following sections discuss evidence in the literature as well as primary data that support a direct role for nAChRs in lung cancer.

## Expression and function of nAChRs in lung cancer

The first hint that nAChRs play a direct role in lung cancer comes from several studies demonstrating nAChR expression in several types of cancers (Table 2). In lung cancer, we have detected the expression of several nAChR subunit genes, in particular *CHRNA3, CHRNA5*, and *CHRNB4* (Improgo et al., 2010). Differences in nAChR gene expression between smokers and non-smokers have also been reported (Lam et al., 2007).

**Table 2:** **Types of cancer cells expressing nAChR subunits**.

**Cancer type**	**nAChR subunits**	**References**
Cervical cancer	α5, α7, α9	Calleja-Macias et al., 2009
Colon cancer	α7	Ye et al., 2004
Leukemia	α2, α3, α5, α6, α7, α9, β2, β4	Sato et al., 1999; Chernyavsky et al., 2009
Lung cancer: NSCLC	α3, α4, α5, α6, α7, α9, β2, β4	West et al., 2003; Tsurutani et al., 2005; Lam et al., 2007; Improgo et al., 2010
Lung cancer: SCLC	α3, α5, α7, α9 β2, β4	Codignola et al., 1994; Song et al., 2003; Improgo et al., 2010
Mesothelioma	α7	Trombino et al., 2004
Medulloblastoma	α7	Siegel and Lukas, 1988
Neuroblastoma	α3, β4	Lukas, 1993

Another line of evidence stems from studies showing that nAChR ligands promote several hallmarks of cancer (Hanahan and Weinberg, 2000; Schuller, 2009). Nicotine induces cell proliferation in lung cancer cells via protein kinase C (Schuller, 1989; Codignola et al., 1994) and Akt (West et al., 2003; Tsurutani et al., 2005) activation. Nicotine's carcinogenic metabolites have also been shown to promote cell proliferation in lung cancer cells via serotonin-induced stimulation of the Raf-1/MAPK/c-myc pathway (Schuller and Orloff, 1998; Jull et al., 2001) and the Akt pathway (West et al., 2003; Tsurutani et al., 2005).

In addition, nicotine has been shown to inhibit apoptosis by phosphorylation of Bcl-2 family members (Jin et al., 2004a). Apoptotic evasion potentially contributes to nicotine-induced chemoresistance (Maneckjee and Minna, 1990, 1994). Similarly, NNK inhibits apoptosis by Bcl-2 phosphorylation (Jin et al., 2004b). Moreover, both nicotine and NNK promote cell survival via the NF-kB pathway (Tsurutani et al., 2005).

Intriguingly, the complete cholinergic system is expressed and functional in lung cells, where ACh acts both as an autocrine and paracrine growth factor (Song et al., 2003; Proskocil et al., 2004). Once activated, this growth loop may provide endogenous mitogenic signaling without further nicotine activation. Such a mechanism may explain residual risk for lung cancer even after smoking cessation.

ACh is also thought to act as a pro-angiogenic signal via autocrine and paracrine signaling in endothelial cells (Heeschen et al., 2002). Nicotine promotes angiogenesis in a PI3-kinase and MAPK-dependent manner (Heeschen et al., 2001). Moreover, nicotine and its metabolite cotinine have been shown to up-regulate the expression of the pro-angiogenic factor VEGF (Conklin et al., 2002).

Many of these cancer-promoting processes are abrogated by pan-nAChR or subtype-selective antagonists. Nicotine-induced cell proliferation via fibronectin up-regulation can be abolished by α-BTx (Zheng et al., 2007). Both α-BTx and the non-selective nAChR inhibitor, mecamylamine, also hinder angiogenic growth *in vitro* (Heeschen et al., 2002). Additionally, nicotine-induced Akt activation and airway cell transformation can be inhibited by the α4β2-selective antagonist, DHβE (West et al., 2003). These observations collectively indicate that many of the above cancer-related processes are mediated by nAChRs.

## CHRNA3/A5/B4 in SCLC

**Figure 1:**
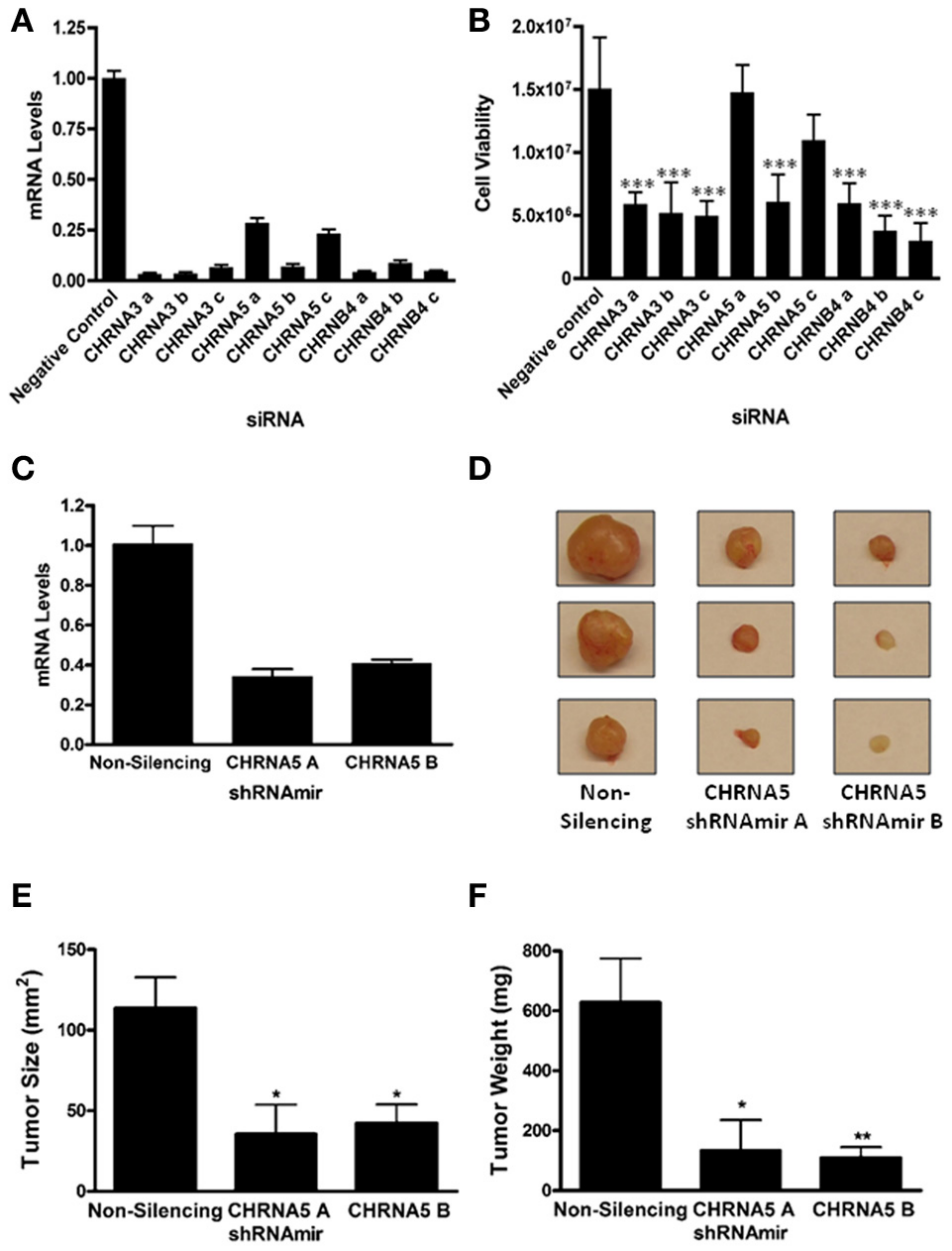
***CHRNA3*, *CHRNA5*, and *CHRNB4* depletion decreases SCLC cell growth**. DMS-53 cells were treated with three distinct *CHRNA3*, *CHRNA5*, and *CHRNB4* siRNAs or a negative control siRNA (Applied Biosystems). **(A)** Quantitative RT-PCR was performed to determine mRNA levels upon knockdown. **(B)** A bioluminescence-based cell viability assay was performed to determine the effect of siRNA treatment on SCLC cell viability (^***^p < 0.001, ANOVA, Tukey *post-hoc* test). **(C)** *CHRNA5* mRNA levels in DMS-53 cells stably expressing a non-silencing shRNAmir or two *CHRNA5* shRNAmirs (Open Biosystems). shRNAmirs were transduced into DMS-53 cells using a lentiviral delivery system for stable expression (Open Biosystems Trans-Lentiral Packaging System). **(D)** DMS-53 cells stably expressing a non-silencing shRNAmir or two CHRNA5 shRNAmirs were subcutaneously injected into the hind flanks of athymic nude mice. Tumors were harvested after 2 months (representative images shown). Tumor size **(E)** and weight **(F)** were significantly lower in samples treated with CHRNA5 shRNAmirs vs the non-silencing control (^*^p < 0.05; ^**^p < 0.01, ANOVA, Tukey *post-hoc* test).

Given the GWAS association between the *CHRNB4/A3/A5* locus and lung cancer risk, we pursued the hypothesis that nAChRs containing the α3, α5, and β4 subunits play a direct role in the development of lung cancer. We focused on SCLC as we had previously observed high expression of *CHRNA5* and upregulation of *CHRNA3* and *CHRNB4* in SCLC (Improgo et al., 2010). We investigated the role of *CHRNA3, CHRNA5*, and *CHRNB4* in SCLC by silencing the expression of these three genes in the SCLC cell line, DMS-53. Three distinct siRNAs against each gene were used (Figure 1A). We assessed the effect of knockdown using a bioluminescence-based cell viability assay as previously described (Improgo et al., 2011). All the siRNAs that yielded > 90% knockdown levels produced corresponding decreases in SCLC cell viability (*n* = 5 each, Figure 1B), suggesting that expression of *CHRNA3*, *CHRNA5*, and *CHRNB4* is vital for SCLC cell viability, at least in the context of the DMS-53 cell line.

The two siRNAs that yielded the least knockdown (CHRNA5 a and c) did not significantly affect cell viability, suggesting that certain *CHRNA5* depletion thresholds may need to be reached to obtain an observable phenotype. To therefore obtain more robust silencing, we utilized shRNAmirs, hairpins that are designed with flanking miRNA sequences that can harness the cell's endogenous RNAi machinery and promote more efficient knockdowns. In addition, we introduced these shRNAmirs to DMS-53 cells via lentiviral delivery to allow stable expression. For a more physiological approach, we used a tumor xenograft model for *in vivo* propagation of tumors. Cells were implanted into immunocompromised mice and tumor growth was monitored. *CHRNA5* shRNAmir A treatment (*n* = 5) caused a 59% decrease in *CHRNA5* levels while *CHRNA5* shRNAmir B treatment (*n* = 9) caused a 66% decrease in mRNA levels (Figure 1C). Quite strikingly, tumor size and tumor weight (Figures 1D–F) were significantly lower in cells treated with the *CHRNA5* shRNAmirs vs. the non-silencing shRNAmir control (*n* = 9). These results further support the *in vitro* data described above.

**Figure 2:**
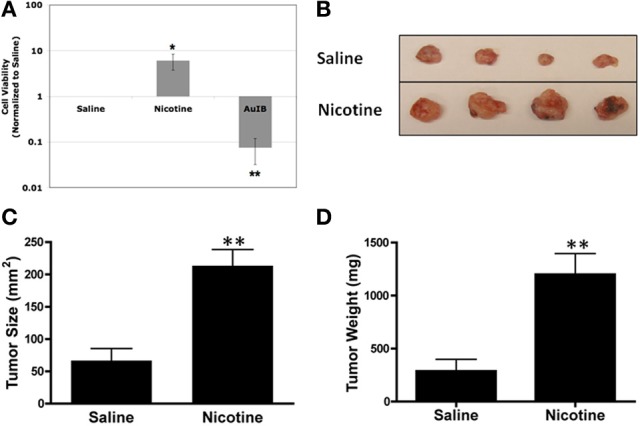
**Pharmacological activation or inhibition of nAChRs modulates SCLC growth. (A)** DMS-53 cells were treated daily for 1 week with 1 μM nicotine or 2 μM α-conotoxin AuIB. Cell viability assays show that nicotine increases while AuIB decreases SCLC cell viability (values normalized to saline control; ^*^p < 0.05, ^**^p < 0.01). **(B)** DMS-53 cells were injected subcutaneously into the hind flanks of athymic nude mice. Mice were then implanted with osmotic minipumps that delivered either saline or 24 mg/kg of nicotine daily. Tumors were harvested after 1 month (representative images shown). **(C,D)**. Chronic nicotine exposure increased both tumor size and weight (^**^p < 0.01, Student's *t*-test).

We next tested the effect of nAChR ligands on SCLC cell viability. Using the same bioluminescence assay as above, we observed that nicotine treatment increased SCLC cell viability (Figure 2A), consistent with aforementioned reports. To perform the converse experiment, we utilized the α3β4-selective ligand, α-conotoxin AuIB. α-conotoxins are derived from the venom of cone snails, a valuable source for disulfide-bonded peptides that target nAChRs in a highly subtype-selective manner (Azam and McIntosh, 2009). α-conotoxin AuIB, in particular, was isolated from the snail-eating cone *Conus aurilicus* and blocks α3β4 nAChRs with > 100-fold higher potency compared to other nAChR subtypes (Luo et al., 1998). Treatment with α-conotoxin AuIB led to decreased viability of DMS-53 cells (Figure 2A), indicating that functional α3β4 nAChRs are present in SCLC cells and are important for the maintenance of SCLC cell viability. In agreement with our genetic approach, this pharmacological approach suggests that activation and blockade of α3α5β4 nAChRs modulates SCLC cell viability.

We then tested the effect of chronic nicotine treatment on tumor growth *in vivo*. We used osmotic minipumps to deliver nicotine as these devices allow continued dosing of drugs while eliminating repeated injections (Salas et al., 2004). Using the same xenograft tumor model as above, we found that chronic nicotine treatment increased tumor size and weight *in vivo* compared to saline controls (Figures 2B–D). This is in line with previous findings showing that nicotine promotes tumor growth in various *in vivo* models (Davis et al., 2009; Al-Wadei et al., 2012).

Our results show that *CHRNA3, CHRNA5*, and *CHRNB4* expression is critical for SCLC cell viability. These findings lend mechanistic support to the correlative link between the *CHRNB4/A3/A5* locus and lung cancer susceptibility. That α3β4α5 nAChRs play a direct role in lung cancer, in addition to their role in the brain, points to the pleiotropic function of these genes. Along with published reports, our work suggests a mechanism by which cholinergic signaling via α3β4α5 nAChRs promotes SCLC growth. Though this may raise questions regarding the use of nicotine-based smoking cessation approaches, it also indicates the potential of nAChR antagonists for SCLC therapy.

### Conflict of interest statement

The authors declare that the research was conducted in the absence of any commercial or financial relationships that could be construed as a potential conflict of interest.

## Abbreviations

NSCLC: non-small cell lung carcinoma
SCLC: small cell lung carcinoma
ACh: acetylcholine
nAChRs: nicotinic acetylcholine receptors
NNK: 4-(methylnitrosamino)-1-(3-pyridyl)-1-butanone
GWAS: genome-wide association studies.
